# Simulation-based research for digital health pathologies: A multi-site mixed-methods study

**DOI:** 10.1177/20552076241247939

**Published:** 2024-05-17

**Authors:** Isabel Straw, Joanna Dobbin, Demelza Luna-Reaver, Leonie Tanczer

**Affiliations:** Institute of Health Informatics, 4919 University College London, London, UK

**Keywords:** Digital health, technology, medicine, emergency medicine, women’s health, connected devices

## Abstract

**Background:**

The advance of digital health technologies has created new forms of potential pathology which are not captured in current clinical guidelines. Through simulation-based research, we have identified the challenges to clinical care that emerge when patients suffer from illnesses stemming from failures in digital health technologies.

**Methods:**

Clinical simulation sessions were designed based on patient case reports relating to (a) medical device hardware errors, (b) medical device software errors, (c) complications of consumer technology and (d) technology-facilitated abuse. Clinicians were recruited to participate in simulations at three UK hospitals; audiovisual suites were used to facilitate group observation of simulation experience and focused debrief discussions. Invigilators scored clinicians on performance, clinicians provided individual qualitative and quantitative feedback, and extensive notes were taken throughout.

**Findings:**

Paired t-tests of pre and post-simulation feedback demonstrated significant improvements in clinician's diagnostic awareness, technical knowledge and confidence in clinical management following simulation exposure (p < 0.01). Barriers to care included: (a) low suspicion of digital agents, (b) attribution to psychopathology, (c) lack of education in technical mechanisms and (d) little utility of available tests. Suggested interventions for improving future practice included: (a) education initiatives, (b) technical support platforms, (c) digitally oriented assessments in hospital workflows, (d) cross-disciplinary staff and (e) protocols for digital cases.

**Conclusion:**

We provide an effective framework for simulation training focused on digital health pathologies and uncover barriers that impede effective care for patients dependent on technology. Our recommendations are relevant to educators, practising clinicians and professionals working in regulation, policy and industry.

## Introduction

A digital technology that breaks while connected to, or implanted in, a patient's body, may initiate a cascade of physiological changes that induce novel symptoms presenting as previously unseen clinical phenomena.^1–3^ The increasingly ubiquitous presence of digital systems in patient care means that clinical phenomena related to technological failures are likely to rise; yet, at present, practitioners are not trained to respond to cases emerging from digital causes (e.g. harm from hacked medical devices a.k.a ‘medjacking’).^1–7^

Technological failures can manifest as adverse clinical events^2^; researchers have described respiratory compromise resulting from malfunctioning vagal nerve stimulators,^8^ cardiac arrests due to faults in ventilators^9^ and patient deaths from errors in diabetic pumps.^10^ A seminal work from Dameff et al. described high-fidelity clinical simulations based on pathologies secondary to ‘hacked’ medical devices, highlighting the risks of compromised bedside infusion pumps, automated internal cardioverter defibrillators, and insulin delivery devices.^1^ Since this research, real-life cases have been reported in the media, including an incident in the UK of a domestic assault enacted via a Bluetooth-connected insulin pump.^11^

In our research on digital health pathologies, we go beyond the domain of medical cybersecurity, and expand our scope to encompass clinical presentations stemming from non-malicious digital events (e.g. unintentional faults in medical devices) and wider consumer technologies that can have health effects (e.g. complications of consumer smart implants).^3^ In considering clinical cases relating to digital technology, we adopt the term ‘biotechnological syndromes’ from a recent systematic review of 372 cases that identified clinical phenomena emerging at the intersection of human health and digital technology.^3^

In the digital fabric of our healthcare institutions, the pathways to digitally mediated harm are diverse and may include devices that directly interface with a patient (e.g. a ventilator) or seemingly distal systems (e.g. cloud-based remote care platforms).^1–6^ At the individual level, cybersecurity researchers have documented extensive vulnerabilities in implantable medical devices, which if exploited have the potential to incur severe morbidity including blindness, paralysis, pain and cardiac arrhythmia.^12–17^ Attacks on the wider medical infrastructure may also cause morbidity and mortality, exemplified by the death of a woman requiring transfer in Dusseldorf due to a hospital cyberattack.^18–20^

Biotechnological syndromes emerge at the intersection of the traditionally siloed domains of clinical medicine, engineering, computer science and cybersecurity.^3^ Thus, understanding how to treat the patient experiencing end-point symptoms of these multifaceted pathologies requires a clinical and technical lens, and a skillset that extends beyond traditional medical training.^1–3^ Currently, a great deal of research into digital health harms focuses on the role of manufacturers and regulatory agencies in improving device oversight, however these interventions can be slow to implement, and need to be paired with changes on the floor of clinical practice.^21–28^ Given that research has demonstrated that 96.6% of reports on regulatory platforms such as FDA MAUDE database are made by manufacturers (as opposed to treating clinicians), it is perhaps unsurprising that a clinical perspective has been lacking when designing a response to these events.^23^ In our research we utilise simulation methods in order to centre the clinician–patient interaction during digital events, and examine the gaps in knowledge, training and resources that impede effective medical care during these scenarios.

Simulation-based research (SBR) has been described as an effective tool for improving quality and safety in healthcare by replicating real-world situations.^29–34^ The improved realism of simulation techniques (e.g. through manikins, actors and props) has facilitated the creation of increasingly complex clinical simulation environments, capable of mimicking real-world scenarios and unearthing real-time challenges that occur during difficult clinical scenarios and in complex patient care.^29–32^ Researchers have detailed the benefit of simulations for observing healthcare teams’ responses to unanticipated crises as they unfold, which is not possible in actual patient care situations and thus offers a powerful tool for improving patient safety and ensuring readiness for rare events (e.g. bioterrorism attacks, natural disasters).^31–33^ Furthermore, simulation has been demonstrated as an effective way for upskilling practitioners in neglected areas of clinical medicine (e.g. transgender healthcare) and in response to changing population health needs (e.g. palliative care).^34,35^ SBR can therefore be a means for identifying barriers to patient care and improving clinical practice in a safe setting.^29–35^ In our research, we explore the value of clinical simulation for improving patient care with regards to adverse digital events.

Our research uses simulation for two purposes:

1. To uncover the key barriers impeding effective patient care in digital clinical cases.
2. To evaluate the value of simulation training for improving the ability of clinicians to respond to digital clinical cases.

We consider issues with both medical devices (including software and hardware issues) and consumer technology. For consumer technology, we focus on the potential implications of smart implants, and the patient safety implications of tracking devices that have been described in cases of technology-facilitated abuse (TFA).^36–39^ Across the four clinical simulation scenarios defined below, we explore the barriers to effective patient care, identify the key challenges faced by clinicians, and explore opportunities for improving future clinical care and ensuring the safe implementation of digital health innovations.

## Methodology

**Figure 1.**
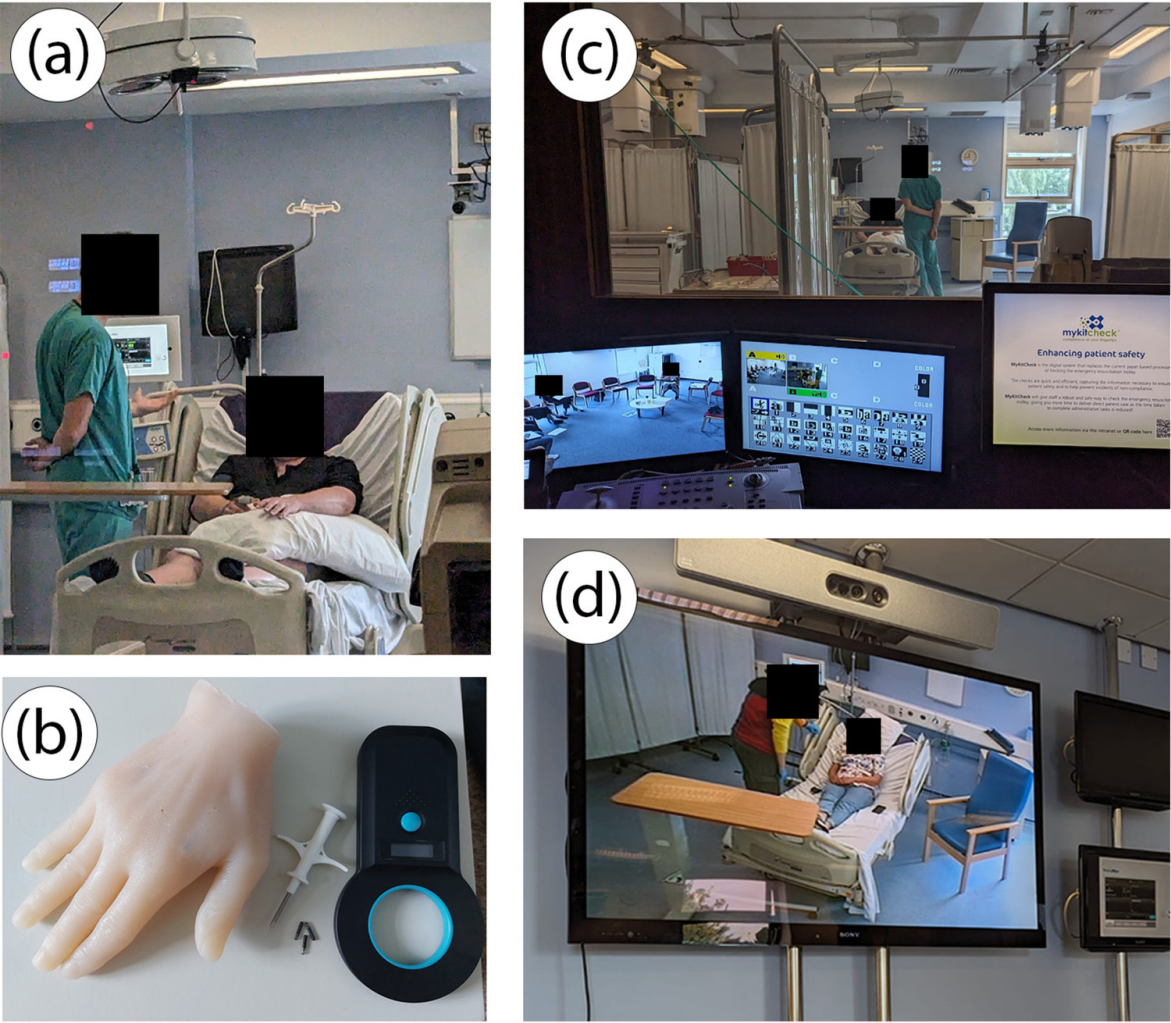
A series of photos demonstrating the set-up of the clinical simulation. The actor and participant are presented in (a), an example of the props used (anatomical hand and RFID chips) in (b), while (c) and (d) demonstrate the use of the simulation suite's AV system. The simulated clinical encounter in (b) was streamed live to the participants in the audience next door (d).

Between 1 February 2023 and 1 July 2023, a series of four simulation sessions were run at three different hospital sites in the United Kingdom. Healthcare staff (nurses, doctors and clinical medical students) were recruited via National Health Service (NHS) Trust emails and advertisement flyers (n = 14). Ethics approval was obtained from University College London (UCL) Research Ethics Committee and all participants provided written informed consent for their contributions to be used in the research study. Sessions were run using simulation suites fitted with audiovisual (AV) capabilities and prototypes of devices were used throughout sessions (Figure 1). The full materials used throughout the simulation sessions, including the scenario descriptions, actor and doctor briefs, and participant feedback forms are provided in the appendices and Supplemental Material to facilitate the reproduction of our work.

### Simulation scenarios

Table 1 provides a summary of the four clinical scenarios that were designed based on existing literature and published case reports.^3,40^ Detailed instructions for actors and sim facilitators were provided for each scenario to ensure consistency across sites. We focused on four different themes of technological failures (full details of scenarios are published in Appendix 1 for reuse):

1. Scenario 1: Software failure in a medical device.
2. Scenario 2: Medical presentation of TFA.
3. Scenario 3: Complication of consumer technology.
4. Scenario 4: Hardware failure in a medical device.

**Table 1.** Clinical simulation scenarios utilised across the four hospital sites (full details of case scenarios can be found in Appendix 1).

Case Scenario Title	Clinical Narrative
1. Taser-induced malfunction in a DBS in a Parkinson's patient	A 54-year-old male presents to the emergency department acutely unwell with dysarthria, choreiform dyskinesia and reporting a severe occipital headache ^40^. The cause stems from a software malfunction in the patient's DBS, which occurred due to electromagnetic interference from a taser that the police had used on the patient.
2. A&E presentation of domestic violence, complicated by TFA	A 33-year-old female, 19 weeks pregnant, presents to the Emergency Department complaining of headache and dizziness following a fall at home. The patient is a victim of domestic abuse, however, is reluctant to engage with services due to the presence of eavesdropping spyware and GPS-tracking apps on her mobile devices.
3. The adolescent basketball player with an RFID microchip	A 16-year-old male presents to his GP accompanied by his mother who states she is very concerned about the ‘Microchip’ in his hand. In this case, the teenager has obtained a subcutaneous RFID chip, his only concern is the impact of the implant on his ability to play basketball.
4. Brachial plexus injury secondary to impingement from an ICD	A 26-year-old female presents with left arm pain and numbness in the median nerve distribution of the left hand. The underlying cause is an impingement from the patient's ICD (previously implanted for Brugada's syndrome) affecting the brachial plexus.

**Table 2.** Details of study participants.

	Seniority	Grade of Doctor (As per the UK NHS Training Scale^30^)	Clinical Specialty
1	Junior	House Officer (F1)	Respiratory Medicine
2	Senior	Consultant	Maxillofacial Surgery
3	Junior	Clinical Medical Student	Medicine
4	Junior	Clinical Medical Student	Healthcare of the Elderly
5	Junior	General Practitioner (ST2)	General Practice
6	Junior	Clinical Medicine Student	Not disclosed
7	Senior	Consultant in Major Trauma	Oral and Maxillofacial Surgery
8	Senior	Senior Registrar (ST4) Doctor	Accident and Emergency
9	Junior	Senior House Officer	Healthcare of Elderly Patients
10	Junior	House Officer (F1)	Geriatrics
11	Junior	Senior House Officer (F3)	Medicine
12	Junior	House Officer (F1)	General Surgery
13	Senior	Senior Registrar	Trauma and Orthopaedics
14	Senior	Clinical Nurse Specialist	Endoscopy

nb. Seniority was determined by training level, such that senior staff comprised those in the second half of their training, for example, senior registrar, and those with training complete, for example, consultants.^30^

### Participant feedback: subjective scores

Simulation sessions ran for half a day and began with an introductory lecture on the topic of biotechnological syndromes, with examples that differed from the clinical sims (e.g. seizures in virtual reality, harm from hacked insulin pumps).^3,10,11^ Participants were asked to reflect on cases in their own practice of patient illnesses stemming from digital technology and engage in group discussion on the topic.

Following this, the simulation sessions began. Participants completed pre-simulation and post-simulation survey questions that evaluated their understanding of syndromes related to technology, focusing on five dimensions of clinical skills: (a) awareness of syndromes, (b) knowledge of syndromes, (c) ability to investigate syndromes, (d) ability to treat syndromes and (e) ability to find appropriate resources. The feedback questionnaires were designed based on existing resources described in the SBR domain, where collecting a combination of qualitative and quantitative data has been demonstrated to facilitate the richest description of the participant's experience.^29–33^ We collected subjective qualitative scores for the five dimensions of clinical skills described above, which have previously been identified as essential elements of clinical care and provided space for participants to add free text comments (see Supplemental Material). To evaluate the significance of any change between the pre- and post-simulation scores, paired t-tests were carried out across the collected data. We produced Bonferroni corrected p-values to account for the multiple comparisons and evaluate for significance.

Participants were also asked to score how relevant they felt biotechnological syndromes were to their clinical practice. Finally, participants completed scenario-specific feedback, in order to facilitate a comparison of the challenges that differed between the various types of technology and clinical domains.

### Examiner marking: objective scores

One participant took part in each clinical scenario, who was scored by one researcher acting as an invigilator. Mark schemes were written to reflect UK medical school ‘Objective structured clinical examinations’ (OSCEs), where each defined point covered an area of clinical competence (e.g. professionalism, clinical examination), for which the participant was awarded a binary score (0/1) depending on their performance and if they achieved the point (mark scheme example and scores provided in Appendix 2).

All participants watched each simulation scenario via AV streaming, which was followed by a 15-minute focused group debrief during which two scribes took extensive notes that were retrospectively analysed through a thematic analysis (Figure 1). The thematic analysis was performed by two members of the research team, who analysed themes that emerged in the qualitative data regarding the key areas of interest set out in the research aims (barriers to effective care and the value of simulation training).

## Results

Our results are divided into (a) scenario-specific findings and (b) cross-scenario findings. The research participants were from a diverse range of disciplinary backgrounds (surgical, hospital medicine and community practice), ranging in seniority from clinical students to hospital consultants. The full details of participants are provided in Table 2, which also provides the breakdown by seniority.^30^ Participants were categorised as either ‘juniors’ (ranging from clinical medical students to junior trainees) or ‘seniors’ (including speciality registrars, general practitioners [GPs] and consultants).^30^

### Scenario-specific findings

The scenarios were intentionally broad as we sought to identify challenges across a range of technologies. Scenario 1 and Scenario 4 both focused on medical devices, one with a software failure and one with a hardware fault. Scenario 2 and Scenario 3 focused on technologies outside of the medical device space, and tested the participants’ knowledge of emerging consumer devices and their ability to consider the wider health implications of these tools.

**Figure 2.**
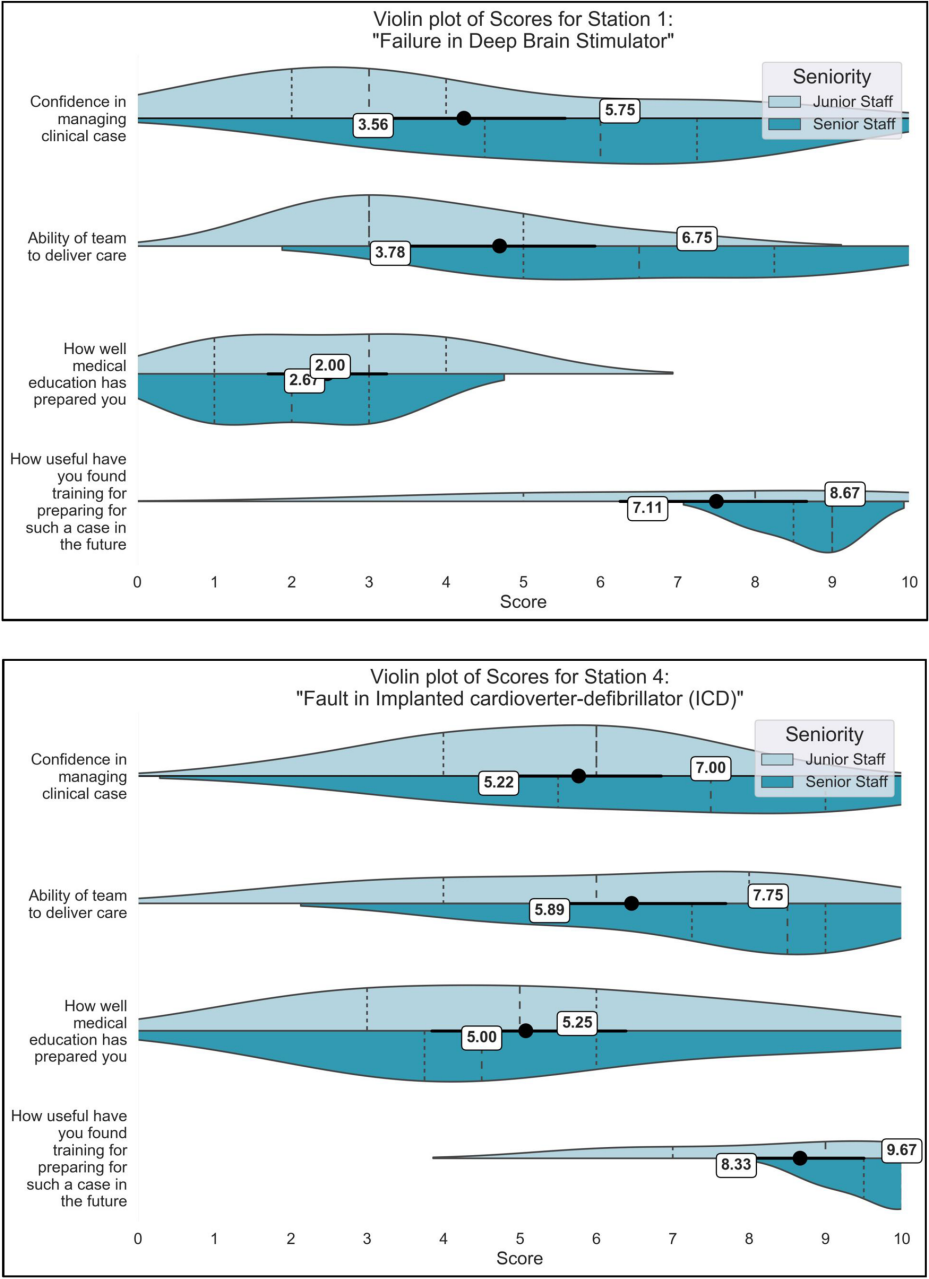
Comparison of Scenario 1 (Software failure in Deep Brain Stimulator) and Scenario 4 (Hardware failure in Implanted cardioverter-defibrillator). Violin plots are split by seniority, such that junior participants (clinical medical students to junior registrars/ST3) and senior participants are visualised side-by-side on plots, with mean scores for each group provided alongside. Significant differences between the junior and senior doctors are marked with asterisks (*).

In the medical device scenarios, participants gave lower confidence scores for managing the software failure, compared to the hardware fault (3.5 vs. 5.2 juniors; 5.75 vs. 7.0 seniors), also reporting that medical education has prepared them less well for the software scenario (2.7 vs. 5.0 juniors, 2.0 vs. 5.25 seniors; Figure 2). One participant described this stating ‘for some reason, I find hardware issues easier to understand/more covered in our training than software’, another shared ‘This case may be less challenging to clinicians as it is a hardware problem which is easier to spot with imaging’. Hardware faults in technology can be picked up on existing radiological modalities and can be understood through the anatomical teaching that medics receive. However, software faults and changes in device settings are defined by shifts in voltage, electrical stimulation and may be influenced by electromagnetic interference, all of which are mechanisms that do not form part of healthcare training.

**Figure 3.**
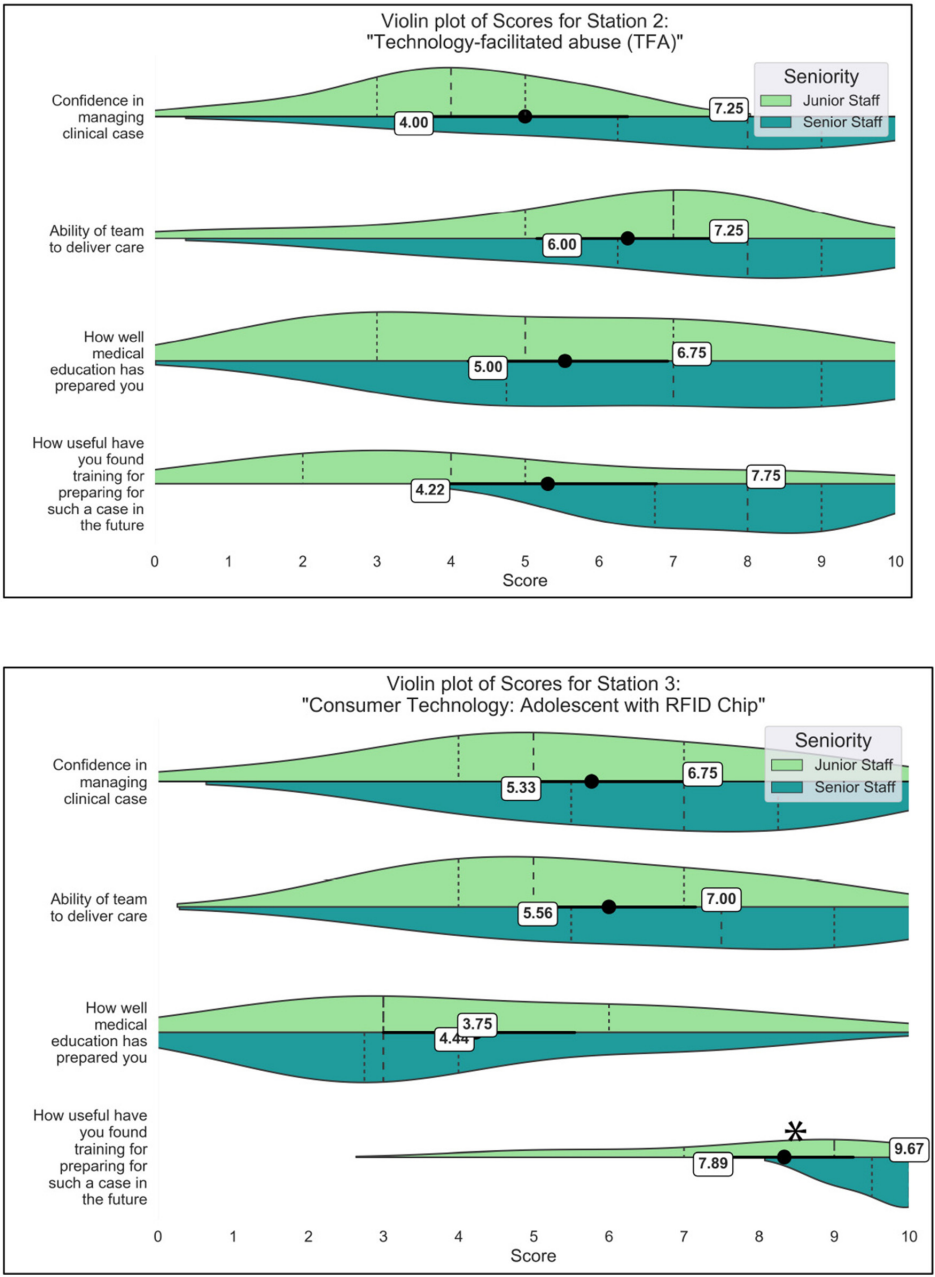
Comparison of survey scores for scenarios involving consumer technologies – Scenario 2 (technology-facilitated abuse involving spyware and GPS tracking) and Scenario 3 (RFID chip implants). Mean scores across all participants (n = 14) are provided for junior and senior doctors separately, for each survey question. Significant differences between the junior and senior doctors are marked with asterisks (*).

Figure 3 provides the participant feedback scores for the scenarios based on technology outside of medical device regulation – Scenario 2 (technology-facilitated abuse) and Scenario 3 (Adolescent with RFID chip). In the tech-abuse scenario, senior participants reported higher confidence in themselves to treat the patient (7.3 seniors vs. 4.0 juniors) and in their teams (7.3 seniors vs. 6.0 juniors) in managing the case. In Scenario 3 with the RFID chip, the gap narrowed for both individual confidence (5.3 juniors vs. 6.8 seniors) and confidence in the team (5.6 juniors, 7.0 seniors). Age and experience may play a role here, such that the more junior participants may be more familiar with the consumer smart implants included in Scenario 3.

### Examples provided by participants

Participants provided examples of biotechnological syndromes they had encountered within their own practice throughout the group discussions and individual feedback, which have been collated in Table 3.

**Table 3.** Examples of biotechnological syndromes provided by participants during group discussions and debriefs.

Quotes From Participants of Examples of Patient Harms Related to Digital Technology
Malfunction of insulin delivery devices – giving wrong doses due to connecting to the wrong handheld device (e.g. smart watch)
We have a problem in the East Midlands *(of the UK)* that we cover a large geographical area. We have a big issue if county lines the young people will be tracked on their phone when they are out on their scooter – their lives are very controlled. They are discharged back to the east coast and it is hard to access these young people – they go through 3 to 4 burner phones or more.
We had a case where a ventilator broke, it was an hour before we figured it out and the patient was properly resuscitated
I have had patients who do not like how they appear on MS Teams/Zoom/Instagram etc, requesting facial surgery to change their appearance despite having a normal appearance
I have seen patients with misfiring ICDs and permanent pacemakers.
Teenagers involved in county lines drug trafficking who are being tracked on burner phones and have no means of escape.
Pacemaker-mediated tachy and bradycardia
Insulin pump malfunction, pacemaker malfunction, cyber abuse and tracking in safeguarding cases
Depression/anxiety from social media
Insulin pump and pacemaker issues. Ventriculoperitoneal (VP) shunt blockage.
We see a lot of GPS tracking of young people involved with county lines

### Cross-scenario findings: barriers to care

Our simulations revealed the challenges that clinicians faced caring for these patients in real-time, aggregated under themes in Table 4. We first consider factors that prevented the identification of the technology as the cause (delays to diagnosis), and then the factors impeding care once the diagnosis was made (delays to clinical management).

**Table 4.** Causes for delay in diagnosis.

Theme	Quotes From Participants
Low suspicion of technology as causal agent	[1] I would not have thought there was a problem, you just always presume the device is working well. [1] It would be very easy to overlook the fact that the patient had a DBS and it was malfunctioning.
Technological causes considered a diagnosis of exclusion	[1] I guess if it was me, I would investigate all organic things before thinking about the DBS. [1] Difficult scenario as a malfunction in the DBS can only be diagnosed by a process of elimination. [4] My first instinct will always be to exclude organic causes due to my medical training.
Attribution to patient mental health	[2] If a patient said their partner knew everything or was tracking them, I had question their mental health. [2] If a patient said I am concerned my partner can read my mind, I would question their mental health rather than their partner's ability to spy on them. [4] ICD Associated perhaps. Although I would also consider it psychosomatic. [2] Patient seemed distracted by her phone? Social media addiction?
Little education on mechanisms of disease	[1] We average doctors do not know the physiology in relation to the technology well enough to work things out, even if we did recognise the problem. [4] We need to move from thinking about pharmacological manipulation of physiology to hardware/software manipulation of bodies. [1] I was trying to figure out how it was controlled, is this because of some sort of interference. [4] In my head, I could not anatomically link the two symptoms. [4] Because he did not have any cardiac symptoms, I did not think it would have anything to do with the ICD. [1] Unsure of the long-term effects of a malfunctioning device for this patient (what is the treatment window?).
Distinguishing device pathology from disease pathology	[1] It is challenging to figure out that there is a tech problem, instead of a physical problem. [1] It is difficult to differentiate between device problems and underlying pathology when they present similarly.
Psychological impact	[1] I am shocked this is even a case that would present, simply because it is so unexpected. [1] Quite scary thinking that this could be an emergency. [1] I just blanked out, first thought to call neuro, I do not know much about DBS.
Issues when patient cannot communicate	[1] With the patient in the state he was in, obtaining a history is difficult. In addition, we have no idea how the device works and so both clinician and patient are in the dark.
Lack of useful clinical investigations	[1] Lab tests and imaging are usually good for excluding things/causes, but not for diagnosing in this case. [4] Even if it is not a software problem and it is an issue where the device migrates, the device analysis might not be able to tell you.

Quotes pertaining to specific scenario feedback are indicated by the number in square brackets (1 = software failure in DBS, 2 = technology-facilitated abuse, 3= adolescent with RFID chip, 4 = hardware failure in implanted cardiac device, 5 = general discussion).

**Table 5.** Barriers to effective treatment.

Theme	Quotes From Participants
Lack of skills amongst healthcare staff	[4] I would want to know who I could talk to about this issue. I do not think my seniors would necessarily be prepared for this either. [4] I feel like if I escalated this, there would be pushback on this as there is a lack of knowledge.
Lack of technical staff in clinical settings	[4] There is not an on-call computer programmer is there – and if there was, would there be one at each trust? [4] No electrophysiologist on call to help with resetting or checking health of device.
Need for support resources	[4] There is almost a need for the equivalent of toxbase for technology. [1] Is there a helpline to call? [1] There is no protocol within the hospital in place for an event of this nature.
Uncertainty regarding follow-up requirements	[5] Whose responsibility is it to report these things? The clinician? [1] If a device malfunctioning, is there somewhere to report things too? [2] How can she be kept safe and where to go on discharge? How can we ensure there is not other trackers?
Hesitation to deliver medical interventions due to lack of knowledge of their clinical effectiveness	[1] Very challenging as conventional medical treatment may not be effective in the case of a malfunctioning device. [3] Unclear guide on how to remove such devices.

Quotes pertaining to specific scenario feedback are indicated by the number in square brackets (1 = software failure in DBS, 2 = technology-facilitated abuse, 3 = adolescent with RFID chip, 4 = hardware failure in implanted cardiac device, 5 = general discussion).

As demonstrated by Table 4, the low suspicion for digital failures led to delay in clinical diagnosis and meant that biotechnological syndromes were approached as a diagnosis of exclusion. In the case of medical devices, participants shared that ‘you just always presume the device is working well’, prohibiting early identification of device issues. In addition, the low suspicion regarding malicious consumer technologies meant that participants did not suspect issues of GPS tracking or eavesdropping in the tech-abuse case. Participants shared that ‘tracking/monitoring devices are very subtle, so easy to miss’ and that the main challenges in the case were ensuring patient safety in the context of malicious surveillance and logistical issues of separating the victim from their devices. Across the four sessions, only one participant removed the devices from the consultation to account for potential listening technologies, an intervention that many felt to be useful but that would be challenging in clinical environments – ‘What's the safest thing to do with a patient's belongings/devices if you think they have eavesdropping technology? Should you move them? Or use white noise/opera music?’

Participants shared difficulties in understanding the mechanisms of disease that underpinned these syndromes, such as in the case of the cardiac device hardware fault manifesting in limb signs from a brachial plexus injury (Scenario 4). The difficulty in connecting seemingly unrelated physiological processes that were mediated by technology, challenged participants in the scenarios of software and hardware failures. Lack of pathophysiological understanding may also have influenced the participant's tendency to attribute symptoms to mental health, such as attributing the device complication to psychosomatic features in Scenario 4. In the tech-abuse scenario, participants reported that they had been more likely to raise concerns regarding a patient's mental health than suspect signs of abuse, with participants linking the increased phone activity to social media addiction, and participants concerns about tracking to their mental health.

The participants described their initial shock when encountering these patients, and a sense of bewilderment at experiencing a clinical presentation that fell outside of their training. Lastly, once participants sought to investigate issues related to the device, they highlighted that their usual repertoire of clinical investigations (e.g. laboratory testing) may be redundant in these cases where syndromes act along different pathophysiological pathways.

### Cross-scenario findings: barriers to effective clinical management

Once participants identified the technology as the source of the problem, further challenges emerged when forming a management plan (Table 5). In particular, both junior and senior staff felt that their team would lack their domain knowledge to treat these cases (Table 5). The gap in skills amongst healthcare staff was felt to be reinforced by a lack of required technical expertise in clinical settings, such as the need for an on-call programmer equipped to reconfigure devices.

When it came to offering treatment in the clinical scenarios, participants were often hesitant due to their uncertainty of how traditional medical treatments would interact with technological processes (e.g. will pharmacological treatments work for pain stemming from a misfiring implanted electrode?). In addition, participants did not want to offer advice on the RFID chip in Scenario 3, due to uncertainty around the technology. Lastly, clinicians were unsure on the pathways for reporting adverse digital events, stating ‘Whose responsibility is it to report these things? The clinician?’

### Suggested interventions for improving patient care

Participants suggested interventions that would have eased the clinical process in these cases, including medical education initiatives (e.g. integrating digital simulations into medical student exams) and the development of clinical support platforms (e.g. a helpline for emergency device failures; see Table 6). The participants also suggested tailored assessments for digital technologies in the clinical workflow, stating that ‘It would be useful to be able to check the integrity of such devices’.

**Table 6.** Suggested interventions for improving future practice provided by participants throughout simulations.

Intervention Suggested	Quote
Medical education initiatives	[1] Some training/teaching on the basics of how to manage symptoms and escalate should be included in teaching. Same way most rare diseases are. [1] Definitely needs to be included in medical curriculum, alongside broad biotechnological syndrome identification and management. [4] Delivering educational sessions to patients on warning signs that their device may be malfunctioning or hijacked. [1] Include in future practice OSCEs. [1] More training, medical education, doctor training regarding hardware/software issues. [1] Education and Sim.
Support platforms	[1] A National resource, e.g. techbase/like tox base. A helpline. [4] ‘Tech base’ – a medical device version of ToxBase
Clinical and digital assessments	[4] It would be useful to be able to check the integrity of such devices (like how we do with observations) before clerking them, to ensure prompt diagnosis.
Staffing needs	[1] Having computer scientists on night shifts. [2] Having a dedicated safeguarding team that specialises in technology abuse and when discharging patients the team would look into how to ensure their home/environment will be free of potential harm from technology.
Hospital protocols and guidelines	[1] Develop guidelines that cover implantable medical devices. [1] Easy to access guidelines. [2] Posters in doctors office/mess to raise awareness (*on tech-abuse).* [2] Have appropriate referral pathways, protocols, systems and infrastructure for scenarios involving tech-abuse and to make doctors aware of it. [2] Install simple mechanisms that will allow patients to open up – banning phones in some consultations. [2] It can be really difficult in GP when doing telephone consultations, if you are worried about tech-abuse from the partner. Sometimes we make up fake appointments in order to see them face-to-face, e.g. say you are booking them in for a cervical smear.
Physical resources	[2] Provide safe place to store mobile devices (if presumed to be bugged) before having clinical consultations. [2] Maybe if equipment was outside rooms/area to take bags to. [2] Potentially removing tech should be a priority whenever asking about domestic abuse. [2] ‘I feel that there isn’t a protocol in place or anywhere we could keep technologies that place the patient at risk whilst we meet the patient’. [2] You could have a written board, with ‘are you feeling safe’ written on it, to avoid speaking (*in the tech-abuse scenario). [2] You could have a print-out in appointments, with the option of devices being removed and written down.

### Examiner marks and value of clinical simulation training

**Figure 4.**
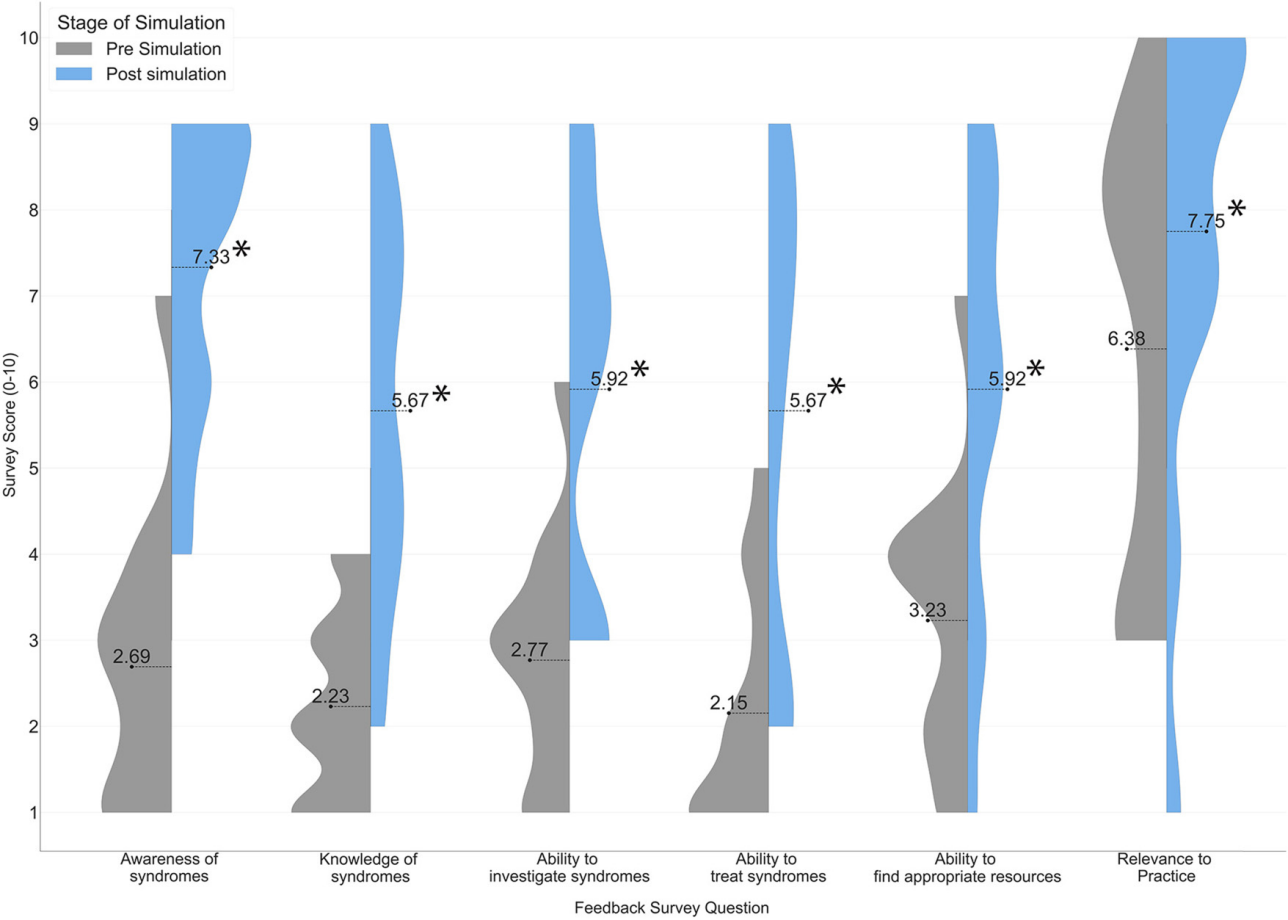
Comparison of participant survey scores before and after clinical simulation, across factors of clinical awareness, knowledge, ability to investigate/treat/find resources and perceived relevance of content (paired t-tests of pre–post scores gives p < 0.01 for all categories, indicated in the Figure with asterisks [*] to illustrate significance).

Simulation training was found to be an effective technique for improving participants’ perceived clinical confidence and knowledge of biotechnological syndromes (Figure 4). Paired t-tests (one-tailed) were performed for all survey scores presented in Figure 4, and across all five questions, there were statistically significant improvements (p < 0.01). These findings are supported by the scores of ‘usefulness’ presented in Figure 2, in which participants consistently rated the usefulness of the session over 8–9/10. Further details on participant mark schemes are available in Appendix 2, which demonstrate participants scored lowest on aspects related to technology in each scenario including (a) managing the digital problem, (b) addresses technical questions and (c) discussion of technology with the patient.

## Discussion

The advance of digital health technologies has created new forms of pathology which are not captured in current medical curricula or clinical guidelines. The growing gap between potential patient pathology and clinical competency is an urgent challenge that must be addressed to ensure patients receive the care they need when they suffer from digital health harms. Our research provides insights into the gaps in current medical domain knowledge, clinical practice and hospital guidelines, and we offer recommendations for practitioners on the frontline and those working in regulation, policy and enterprise.

### Recommendation1: development of resources

Participants described the need for national helplines and a database of biotechnological pathologies, in particular, an app that lists the potential side-effects of all medical devices would be useful. Such a helpline has been proposed before, with the database requiring an interface that is easily accessible and extracts only the most clinically relevant information regarding device failures, so as to avoid clinicians needing to search through individual medical device manuals which are often verbose and dense.^41^ The participants also suggested integrating an evaluation of digital devices into the clinical workflow, to ensure digital errors were picked up at the point of patient clerking. These suggestions speak to the wider research on the effective integration of digital technologies within the NHS, from emergency medicine to digital pathology.^21,42^ In addition, clinicians requested safeguarding guidelines specifically tailored to TFA, that could provide standardised measures for protecting patients in suspected cases of harm.^43,44^ These guidelines may introduce generic measures such as removing potentially ‘listening’ devices from a consultation room and the use of Faraday bags if there are concerns of geolocation tracking.

### Recommendation 2: future research into biotechnological syndromes

When faced with a digital pathology, participants consistently reported ruling out ‘organic’ causes first and raised concerns about not having the tests to identify biotechnological issues. If participants were trained in the halo signs of biotechnological issues, such as respiratory symptoms being indicative of device malfunction in VNS patients, these biotechnological diagnoses would not need to wait for exclusion. To improve the diagnostic capability of clinicians, further research is needed into the onset symptoms and signs of these syndromes. In addition, there is a lack of research on the effective symptomatic management of physiological issues associated with technological complaints, for example, are pharmacological interventions as effective for pain stemming from faults in implantables (e.g. a malfunctioning deep brain stimulator [DBS]^40^). The growing research that explores the benefits of new medical devices needs to be paired with an equally deep investigation into their potential long-term healthcare implications, the novel pathological pathways that emerge with new implants and the treatments that are effective when they go wrong.

### Recommendation 3: education, clinical training and simulation

The scientific language of the life sciences and the physical sciences has historically developed along separate trajectories. However now that human patients depend on physical-digital devices, understanding their health needs requires an interdisciplinary knowledge that intersects these domains. Similarly to the means by which pharmacology became embedded in medical school curricula, clinical students would benefit from basic training in medical technology, covering the foundational components of these tools and the forces by which they function and may fail. AI researchers have advocated for the integration of AI competency training for clinicians in medical schools, we propose that this should be expanded to encompass the wider digital health issues we have discussed including topics such as hardware and software failures, and cybersecurity exploits.^43^ Further, we have demonstrated the value of utilising simulation-based methods for this purpose. Our results demonstrate significant improvements in the awareness and confidence of clinicians pertaining to these cases following a half-day exposure to digital health simulations.

### Health equity

The burden of digital health complaints may differ between demographic groups. In discussions of TFA, participants raised concerns that some patients may be more at risk including patients with dementia who may be subject to elder abuse and young people at risk of knife crime and gang violence.^36–39^ Participants also shared concerns about the RFID chip technology that could be imposed on groups without consent, and given the increased investment in smart implant technologies with growing functional capabilities, these may hold significant threats to privacy and bodily autonomy in the future.

Urban–rural health disparities came up as an issue, as participants identified that non-specialist centres were less likely to have required expertise, for example, an out-of-hours implantable cardioverter defibrillator (ICD) expert. Our increasing reliance on digital infrastructure may exacerbate urban–rural health disparities. Furthermore, we noted that clinicians had a tendency to ascribe confusing presentations to psychosomatic issues and mental health complaints. Previous research has demonstrated that these biases often fall along racial and gender lines, such that there is often a delay in investigating and diagnosing illnesses affecting women, and racial and ethnic minorities.^44–50^ The announcement from the UK Government to deliver an independent review into the equity of medical devices is a welcome initiative for exploring these issues, and further research into these issues specifically within biotechnological syndromes would be welcome.^50^

### Limitations

Our study is subject to selection bias given that the participants volunteered for the session based on our recruitment email, and we are limited by a low sample size (n = 14). Furthermore, all sites were based in metropolitan areas and therefore we cannot account for differences in urban to rural hospital settings. Lastly, our small sample size (n = 14) restricted our statistical analysis. Identifying significant differences between the junior and senior participants was particularly challenging, as this required stratification into even small subgroups. Further research that replicates our work with larger sample sizes would be beneficial for fully evaluating these effects.

## Conclusion

The gap between forms of patient pathology, and clinical understanding, is growing. The new digital technologies woven into both our society and our bodies are changing the way in which we experience health and disease, creating new clinical pictures that require tailored clinical support built from a foundation of digital understanding. In our research we have demonstrated the value of SBR for uncovering barriers to care and latent threats to patient safety that exist in digital clinical scenarios. In addition to demonstrating the value of clinical simulation training, we provide a series of recommendations for clinical practice for improving patient care and ensuring institutional readiness for the rising tide of digital health pathologies.

## Supplemental Material

sj-docx-1-dhj-10.1177_20552076241247939 - Supplemental material for Simulation-based research for digital health pathologies: A multi-site mixed-methods study Supplemental material, sj-docx-1-dhj-10.1177_20552076241247939 for Simulation-based research for digital health pathologies: A multi-site mixed-methods study by Isabel Straw, Joanna Dobbin, Demelza Luna-Reaver and Leonie Tanczer in DIGITAL HEALTH

sj-docx-2-dhj-10.1177_20552076241247939 - Supplemental material for Simulation-based research for digital health pathologies: A multi-site mixed-methods study Supplemental material, sj-docx-2-dhj-10.1177_20552076241247939 for Simulation-based research for digital health pathologies: A multi-site mixed-methods study by Isabel Straw, Joanna Dobbin, Demelza Luna-Reaver and Leonie Tanczer in DIGITAL HEALTH

## Appendix 1. Clinical scenarios delivered at each site, with references to case reports from which medical information was derived.

### Scenario 1 – Taser-induced malfunction in a DBS in a Parkinson's patient

#### Background

The case is based on a patient with a DBS failure, in which incorrect voltage settings caused uncontrollable tremor, involuntary movements and dysarthria. The cause of the malfunction remains unclear. The clinical literature provides some information on possible causes of DBS failure, such electromagnetic (EM) interference from sources including security gates and tasers.

In this case, we consider a taser to be the source of EM interference. Tasers are a digital technology which can induce harm themselves and hence act as an independent digital player. Taser's have been demonstrated to cause facial trauma,^1^ scarring alopecia^2^ and kidney injury with rhabdomyolysis.^3^ In this case, we incorporated digital elements at each stage of the patient journey:

1. The patient's initial presentation of psychosis may itself have related to a DBS error (common in end of battery life pathology).
2. The deployment of the Taser by law enforcement may have caused EM interference affecting the DBS function causing the shift to dyskinesia.
3. The abnormal laboratory findings of acute kidney injury may relate to either the Taser, the dyskinesia from the failing DBS or both.
4. Full resolution of symptoms requires the presence of a programmer.

#### Case

A 54M presents to the emergency department acutely unwell with dysarthria, choreiform dyskinesia and reporting a severe occipital headache. The patient is accompanied by two police officers who apprehended the patient in the community due to aggressive behaviour. The police had been called to a local Tesco where the patient appeared erratic, stated that the food on the shelves were poisoned and began pulling down the shelves. Due to an escalation in violence, the police officers deployed a Taser, at which point the patient began ‘violently shaking and has not stopped since’. The patient has a history of hypothyroidism, Parkinson's disease and depression, with a DBS implanted 6 years earlier.

#### Resources for case

- DBS patient programmer: Picture of remote control present in patient's bag.
- Taser: details of Taser model deployed by police.

#### Learning points

Electromagnetic interference: Discuss the range of EM interference with DBS including home technologies and tasers.

Clinical syndromes: Discuss the range of symptoms that both DBS errors and taser-induced harms can present with.

Investigations: Discuss clinical investigations that should be included and avoided.

Potential injuries caused by tasers: Discussion of variety of injuries that could be seen in a patient that has been subjected to taser use

### Scenario 2 – A&E presentation of domestic violence, complicated by technology-facilitated abuse

#### Background

Technology-facilitated abuse describes the misuse of digital systems such as smartphones or other Internet-connected devices to harm individuals. In this case, a patient experiencing domestic violence presents to the hospital, and it is the role of the practitioner to unearth the breadth of risk posed to this patient. Newer forms of technology-facilitated harm that healthcare practitioners may not be aware of include the manipulation of smart thermostats, air conditioning systems and music speakers to cause victims harm and impose distress. Furthermore, when creating a safety plan for this patient (e.g. referring to refuge or signposting to resources), the practitioner should be aware of the risks of device-surveillance, GPS tracking and voice-activated surveillance of mobile phones. Participants may also consider demographic features (i.e. high income indicates higher likelihood of home-smart devices).

#### Case

A 33F, 19 weeks pregnant, presents to the Emergency Department complaining of headache and dizziness following a fall at home. The patient states she has been experiencing syncopal symptoms with the pregnancy and relates this to the increased vomiting that she tends to experience in the first trimester. When falling, she has impacted the bridge of her nose and right cheekbone. She is requesting an X-ray to exclude any fractures. When she walks in you observe several bruises on her forearms, and she appears nervous, throughout the consultation her phone buzzes.

#### Learning points

The patient's presentation was related to an episode of domestic abuse in the home, which has escalated since her pregnancy. If the healthcare practitioner asks, they will note two previous presentations for the same issues in her last pregnancy. The patient only discloses the abuse and her concerns once devices are outside the room, and she is no longer concerned about being ‘tracked’ – she may indicate this by asking to put her phone outside the room. The practitioner should consider:

- GPS tracking.
- Details to elicit on history-taking: Smart locks and smart thermostat in the house.
- Electronic surveillance: Phone on the table infected with spyware.
- Risk factors and signs of domestic abuse in the emergency department.

### Scenario 3 – The adolescent basketball player with an RFID microchip

#### Background

The increasing implantation of new technologies into the bodies has potentiated a range of previously unseen clinical phenomena. Beyond the healthcare sphere, new initiatives in the citizen biohacking and consumer industry have included the uptake of radio-frequency identification (RFID) technology.^8^ Since 1998, RFID chips have been implanted in humans, largely by hobbyists and even offered by some employers for uses ranging from access to emergency medical records to entry to secured workstations.^8^ These implanted devices can enable seamless interaction with the Internet of Things (IoT) devices in our environment, for example, unlock doors with a wave of your hand, start your car, or even pay for goods.

#### Case

A 16M presents to his GP accompanied by his mother who states she is very concerned about the ‘Microchip’ in his hand. The patient's mother reports she saw a Facebook post in which the patient was having a microchip placed in the first webspace of the right hand, which can be felt below the skin. She would like your advice regarding the risks of the technology, such as whether it can cause cancer, and if you can take it out. On discussing the chip with the patient, the patient states that he does not want to have it removed however he would like to know whether he can keep playing basketball for his school team. What advice and management would you offer this patient?

#### Resources

Information leaflet: The patient's mother has brought an information leaflet from the website that offers RFID chips.

X-ray: Available on request.

### Scenario 4 – Brachial plexus injury secondary to impingement from an ICD

#### Background

One challenge for practitioners managing biotechnological syndromes is that the illnesses that manifest within their specialty, may result from a technology unfamiliar to them, initiated within a different disciplinary domain. For example, (a) refractory sexual arousal subsequent to a neurostimulator,^4^ (b) new onset tinnitus following spinal stimulator implantation^5^ and (c) abdominal spasms resulting from pacemaker phrenic nerve stimulation.^6^ In this case, we focus on an unusual ICD complication, in which the impingement of an ICD loop causes a brachial plexus injury.^7^ Through this case, we aim to widen the practitioner's diagnostic perspective to include device-related causes that may originated in a different bodily system.

#### Case

A 26F presents with left arm pain and numbness in the median nerve distribution of the left hand. She reports this pain (now rated 7/10) has come on gradually over the past two weeks and is now preventing her from completing her daily tasks including cooking, cleaning and typing. Aside from the arm symptoms, she is systemically well with no additional symptoms. Her regular medications include vitamin D and iron supplements. She has an ICD that was placed 5 years earlier following a sudden cardiac arrest thought to be related to Brugada's syndrome.

#### Learning points

On examination, the clinician will note a positive Tinel's sign over the ICD pocket. If the clinician attempts to deactivate the device with a magnet, this will not resolve the symptoms. On performing the CT scan, the findings reveal impingement from the ICD loop. On examination, they have reduced sensation over the anterolateral aspect of the hand with reduced power of thumb flexion and wrist flexion.

**References for clinical scenarios**

1. Campbell F and Clark S. Penetrating facial trauma from a Taser barb. Br J Oral Maxillofac Surg 2019; 57(2): 188–189.
2. Abada H, Aktouf A, Delaunay F, et al. Alopecia reconstruction by expansion after a scalp burn injury caused by Taser®: a case report. Ann Dermatol Venereol 2014; 141(12): 769–772.
3. Gleason JB and Ahmad I. TASER® electronic control device-induced rhabdomyolysis and renal failure: a case report. J Clin Diagn Res 2015; 9(10): HD01–HD02.
4. Zoorob D, Deis AS and Lindsay K. Refractory sexual arousal subsequent to sacral neuromodulation. Case Rep Obstet Gynecol 2019; 2019: 7519164.
5. Golovlev AV and Hillegass MG. New onset tinnitus after high-frequency spinal cord stimulator implantation. Case Rep Anesthesiol 2019; 2019: 5039646.
6. Dalex M, Malezieux A, Parent T, et al. Phrenic nerve stimulation, a rare complication of pacemaker: a case report. Medicine (Baltimore) 2021; 100(11): e25060.
7. Jumper N, Radotra I, Witt P, et al. Brachial plexus impingement secondary to implantable cardioverter defibrillator: a case report. Arch Plast Surg 2019; 46(6): 594–598.
8. Fram BR, Rivlin M and Beredjiklian PK. On emerging technology: what to know when your patient has a microchip in his hand. J Hand Surg 2020; 45(7): 645.

## Appendix 2. Mark scheme used by examiners during each clinical scenario. Mark schemes were completed at each NHS hospital site, and averages were taken across the four sites to give the average scores for each mark scheme component that is presented in the research paper.

**Table:**

		Binary Score of 0/1 (1 Indicates Participant Achieved Point)			
Mark Scheme Component	Marking Detail	Scenario 1: Software Malfunction in DBS	Scenario 2: Technology-Facilitated Abuse (TFA)	Scenario 3: Adolescent with RFID Chip	Scenario 4: Hardware Fault in ICD
		Average score across four sites (standard deviation)	Average score across four sites (standard deviation)	Average score across four sites (standard deviation)	Average score across four sites (standard deviation)
Professionalism	Participant treats the patient professionally and with respect	1.0 (0.0)	1.0 (0.0)	1.0 (0.0)	0.6 (0.0)
History taking	Participant asks appropriate questions and elicits history from the patient	1.0 (0.0)	0.5 (0.5)	1.0 (0.0)	0.6 (0.0)
Clinical examination	Participant undertakes relevant examination of the patient	0.3 (0.4)	0.8 (0.4)	0.5 (0.5)	0.5 (0.4)
Identification of technology	Participant identifies the presence of the digital technology	1.0 (0.0)	0.5 (0.5)	1.0 (0.0)	0.6 (0.0)
Clinical diagnosis	Participant identifies the digital technology as the cause of the patient illness	0.5 (0.5)	0.0 (0.0)	1.0 (0.0)	0.4 (0.4)
Discussion of technology	Participant discusses the underlying issues with the digital technology (i.e. EM interference)	0.3 (0.4)	0.0 (0.0)	1.0 (0.0)	0.3 (0.0)
Treats medical symptoms	Participant provides effective medical care (e.g. to treat symptoms)	0.0 (0.0)	1.0 (0.0)	1.0 (0.0)	0.4 (0.0)
Manages digital problem	Participant provides care to resolve the issue with the digital technology (i.e. contacting the manufacturer/programmer)	0.5 (0.5)	0.0 (0.0)	0.3 (0.4)	0.3 (0.0)
Addresses medical questions	Participant is able to address the patients’ medical questions	0.5 (0.5)	0.8 (0.4)	0.0 (0.0)	0.4 (0.4)
Addresses technical questions	Participant is able to address patient's questions about the digital technology	0.0 (0.0)	0.0 (0.0)	0.0 (0.0)	0.0 (0.0)
