# Supplementary material for: Simulation-based research for digital health pathologies: A multi-site mixed-methods study
Source: Digit Health. 2024 May 17;10:20552076241247939. doi: 10.1177/20552076241247939 (PMC11102683; doi:10.1177/20552076241247939)
Supplement: sj-docx-1-dhj-10.1177_20552076241247939 - Supplemental material for Simulation-based research for digital health pathologies: A multi-site mixed-methods study [file sj-docx-1-dhj-10.1177_20552076241247939.docx]

Participant Feedback Form

**Title of Study:** Clinical Training in Medical Cyber-Crises & Biotechnological Syndromes

**Project ID number:** UCL/CSREC/R/18

All information provided on this form adheres to strict ethical standards and will be treated confidentially and anonymously.

1. **Pre-simulation questions**

What is your clinical role, specialty, and level of seniority?

|  |
| --- |

For this study, we define biotechnological syndromes as those arising at the intersection of health and technology. In these syndromes, the patient presentation is related to an interaction with a digital technology.

**Please rank on the following on a scale of 1 (very low) to ten (very high):**

Please rank your **awareness** of the range of biotechnological syndromes that exist and how they may impact patients?

| 1 | 2 | 3 | 4 | 5 | 6 | 7 | 8 | 9 | 10 |
| --- | --- | --- | --- | --- | --- | --- | --- | --- | --- |

How confident do you feel in your **knowledge** of biotechnological syndromes?

| 1 | 2 | 3 | 4 | 5 | 6 | 7 | 8 | 9 | 10 |
| --- | --- | --- | --- | --- | --- | --- | --- | --- | --- |

How confident do you feel in your **ability to investigate** a patient experiencing a biotechnological syndrome?

| 1 | 2 | 3 | 4 | 5 | 6 | 7 | 8 | 9 | 10 |
| --- | --- | --- | --- | --- | --- | --- | --- | --- | --- |

How confident do you feel in your **ability to treat** a patient experiencing a biotechnological syndrome?

| 1 | 2 | 3 | 4 | 5 | 6 | 7 | 8 | 9 | 10 |
| --- | --- | --- | --- | --- | --- | --- | --- | --- | --- |

How confident do you feel in your **ability to find the appropriate resources for** a patient experiencing a biotechnological syndrome?

| 1 | 2 | 3 | 4 | 5 | 6 | 7 | 8 | 9 | 10 |
| --- | --- | --- | --- | --- | --- | --- | --- | --- | --- |

How **relevant** do you think Biotechnological syndromes are to your current clinical practice?

| 1 | 2 | 3 | 4 | 5 | 6 | 7 | 8 | 9 | 10 |
| --- | --- | --- | --- | --- | --- | --- | --- | --- | --- |

Can you think of a biotechnological syndrome that you’ve encountered in your work?

|  |
| --- |

**Scenario 1**

**Please rank on the following on a scale of 1 (very low) to ten (very high):**

How confident would you feel managing this clinical case?

| 1 | 2 | 3 | 4 | 5 | 6 | 7 | 8 | 9 | 10 |
| --- | --- | --- | --- | --- | --- | --- | --- | --- | --- |

How well you think yourself and your clinical team would be able to appropriately deliver care for this patient?

| 1 | 2 | 3 | 4 | 5 | 6 | 7 | 8 | 9 | 10 |
| --- | --- | --- | --- | --- | --- | --- | --- | --- | --- |

How well has your medical education and clinical training prepared you for managing this clinical case?

| 1 | 2 | 3 | 4 | 5 | 6 | 7 | 8 | 9 | 10 |
| --- | --- | --- | --- | --- | --- | --- | --- | --- | --- |

Do you have any initial thoughts regarding this clinical case?

|  |
| --- |

What do you think are the main challenges in providing effective care for this patient?

|  |
| --- |

Are you aware of guidelines in place that would assist you in managing this clinical case?

|  |
| --- |

How do you think we can ensure the best care for a patient in this situation in the future?

|  |
| --- |

How useful have you found this training for preparing you for such a case in the future?

| 1 | 2 | 3 | 4 | 5 | 6 | 7 | 8 | 9 | 10 |
| --- | --- | --- | --- | --- | --- | --- | --- | --- | --- |
|  |  |  |  |  |  |  |  |  |  |

**Scenario 2**

**Please rank on the following on a scale of 1 (very low) to ten (very high):**

How confident would you feel managing this clinical case?

| 1 | 2 | 3 | 4 | 5 | 6 | 7 | 8 | 9 | 10 |
| --- | --- | --- | --- | --- | --- | --- | --- | --- | --- |

How well you think yourself and your clinical team would be able to appropriately deliver care for this patient?

| 1 | 2 | 3 | 4 | 5 | 6 | 7 | 8 | 9 | 10 |
| --- | --- | --- | --- | --- | --- | --- | --- | --- | --- |

How well has your medical education and clinical training prepared you for managing this clinical case?

| 1 | 2 | 3 | 4 | 5 | 6 | 7 | 8 | 9 | 10 |
| --- | --- | --- | --- | --- | --- | --- | --- | --- | --- |

How confident do you feel in logging patient records confidentially when the patient's records may be sought or monitored by a perpetrator of abuse?

| 1 | 2 | 3 | 4 | 5 | 6 | 7 | 8 | 9 | 10 |
| --- | --- | --- | --- | --- | --- | --- | --- | --- | --- |

How comfortable do you feel developing a safety plan that considers technology abuse with this patient?

| 1 | 2 | 3 | 4 | 5 | 6 | 7 | 8 | 9 | 10 |
| --- | --- | --- | --- | --- | --- | --- | --- | --- | --- |

Do you have any initial thoughts regarding this clinical case?

|  |
| --- |

What do you think are the main challenges in providing effective care for this patient?

|  |
| --- |

What could your workplace do to better support you to provide effective care for this patient?

|  |
| --- |

Are you aware of relevant third-party referral pathways that can support the patient with domestic violence technology abuse?

|  |
| --- |

Do you have resources available to you should you during a consultation if you have questions on how to manage this clinical case

|  |
| --- |

How do you think we can ensure the best care for a patient in this situation in the future?

|  |
| --- |

How useful have you found this training for preparing you for such a case in the future?

| 1 | 2 | 3 | 4 | 5 | 6 | 7 | 8 | 9 | 10 |
| --- | --- | --- | --- | --- | --- | --- | --- | --- | --- |

**Scenario 3**

**Please rank on the following on a scale of 1 (very low) to ten (very high):**

How confident would you feel managing this clinical case?

| 1 | 2 | 3 | 4 | 5 | 6 | 7 | 8 | 9 | 10 |
| --- | --- | --- | --- | --- | --- | --- | --- | --- | --- |

How well you think yourself and your clinical team would be able to appropriately deliver care for this patient?

| 1 | 2 | 3 | 4 | 5 | 6 | 7 | 8 | 9 | 10 |
| --- | --- | --- | --- | --- | --- | --- | --- | --- | --- |

How well has your medical education and clinical training prepared you for managing this clinical case?

| 1 | 2 | 3 | 4 | 5 | 6 | 7 | 8 | 9 | 10 |
| --- | --- | --- | --- | --- | --- | --- | --- | --- | --- |

Do you have any initial thoughts regarding this clinical case?

|  |
| --- |

What do you think are the main challenges in providing effective care for this patient?

|  |
| --- |

Are you aware of guidelines in place that would assist you in managing this clinical case?

|  |
| --- |

How do you think we can ensure the best care for a patient in this situation in the future?

|  |
| --- |

How useful have you found this training for preparing you for such a case in the future?

| 1 | 2 | 3 | 4 | 5 | 6 | 7 | 8 | 9 | 10 |
| --- | --- | --- | --- | --- | --- | --- | --- | --- | --- |

**Scenario 4**

**Please rank on the following on a scale of 1 (very low) to ten (very high):**

How confident would you feel managing this clinical case?

| 1 | 2 | 3 | 4 | 5 | 6 | 7 | 8 | 9 | 10 |
| --- | --- | --- | --- | --- | --- | --- | --- | --- | --- |

How well you think yourself and your clinical team would be able to appropriately deliver care for this patient?

| 1 | 2 | 3 | 4 | 5 | 6 | 7 | 8 | 9 | 10 |
| --- | --- | --- | --- | --- | --- | --- | --- | --- | --- |

How well has your medical education and clinical training prepared you for managing this clinical case?

| 1 | 2 | 3 | 4 | 5 | 6 | 7 | 8 | 9 | 10 |
| --- | --- | --- | --- | --- | --- | --- | --- | --- | --- |

Do you have any initial thoughts regarding this clinical case?

|  |
| --- |

What do you think are the main challenges in providing effective care for this patient?

|  |
| --- |

Are you aware of guidelines in place that would assist you in managing this clinical case?

|  |
| --- |

How do you think we can ensure the best care for a patient in this situation in the future?

|  |
| --- |

How useful have you found this training for preparing you for such a case in the future?

| 1 | 2 | 3 | 4 | 5 | 6 | 7 | 8 | 9 | 10 |
| --- | --- | --- | --- | --- | --- | --- | --- | --- | --- |

**Post simulation questions**

Thank you for taking part in our simulation training. Firstly, please feel free to provide any comments on the overall experience:

|  |
| --- |

**Please rank on the following on a scale of 1 (very low) to ten (very high):**

Please rank your **awareness** of the range of biotechnological syndromes that exist and how they may impact patients?

| 1 | 2 | 3 | 4 | 5 | 6 | 7 | 8 | 9 | 10 |
| --- | --- | --- | --- | --- | --- | --- | --- | --- | --- |

How confident do you feel in your **knowledge** of biotechnological syndromes?

| 1 | 2 | 3 | 4 | 5 | 6 | 7 | 8 | 9 | 10 |
| --- | --- | --- | --- | --- | --- | --- | --- | --- | --- |

How confident do you feel in your **ability to investigate** a patient experiencing a biotechnological syndrome?

| 1 | 2 | 3 | 4 | 5 | 6 | 7 | 8 | 9 | 10 |
| --- | --- | --- | --- | --- | --- | --- | --- | --- | --- |

How confident do you feel in your **ability to treat** a patient experiencing a biotechnological syndrome?

| 1 | 2 | 3 | 4 | 5 | 6 | 7 | 8 | 9 | 10 |
| --- | --- | --- | --- | --- | --- | --- | --- | --- | --- |

How confident do you feel in your **ability to find the appropriate resources for** a patient experiencing a biotechnological syndrome?

| 1 | 2 | 3 | 4 | 5 | 6 | 7 | 8 | 9 | 10 |
| --- | --- | --- | --- | --- | --- | --- | --- | --- | --- |

How **relevant** do you think Biotechnological syndromes are to your current clinical practice?

| 1 | 2 | 3 | 4 | 5 | 6 | 7 | 8 | 9 | 10 |
| --- | --- | --- | --- | --- | --- | --- | --- | --- | --- |

Can you think of a biotechnological syndrome that you’ve encountered in your work?

|  |
| --- |

Any final comments

|  |
| --- |
