# Supplementary material for: Simulation-based research for digital health pathologies: A multi-site mixed-methods study
Source: Digit Health. 2024 May 17;10:20552076241247939. doi: 10.1177/20552076241247939 (PMC11102683; doi:10.1177/20552076241247939)

**Clinical Training in Medical Cyber-Crises & Biotechnological Syndromes**

# Scenario 1 Instructions for doctor

## **Summary**

You are about to see a patient who has been brought to the emergency department by the police. The police state that they were called to Tesco’s, where the patient was reportedly ‘erratic’ and causing damage inside the shop. Since arriving to the Emergency Department, the patient appears to have become more unwell, and you have been asked to see them.

## **Instructions to doctor**

- Take a history and examine the patient to establish the cause of his current symptoms
- Form a plan for appropriately investigating and managing the patient

# Scenario 1 Instructions for ACTORS

## Actor 1 = Patient

## Actor 2 = Patient’s partner

## Summary

**Patient:** You are a 54-year-old male patient who has is experiencing violent shaking of your limbs and you are struggling to speak due to the shaking affecting your jaw – your words are coming out in a stammer. You’ve not been feeling well since this morning and had gone to Tesco to do some shopping, but you started to feel angry and distressed – you’re not sure why.

The police came and started bothering you, and when you tried to leave, they deployed a taser. The taser hit your upper back and since this happened all your limbs have been shaking. You feel very tense and rigid, and your primary concern is that you can’t stop this shaking. Aside from the shaking, you have a moderate headache which started this morning (5/10 severity) but no other symptoms.

You have a past medical history of Hypothyroidism, Parkinson’s Disease and Depression, with a Deep Brain Stimulator (DBS) implanted six years earlier. You don’t know much about the (DBS), just that the GP recommended it and it’s helped with your Parkinson’s symptoms of rigidity and tremor.

Your partner who you live with has come to the hospital with you, they were called by the police in Tesco.

**Patient’s Partner:** You are the patient’s partner and noted that the patient was acting strangely since this morning. He was very irritable, seemed in a bad mood, and left early to go shopping. At lunch time, you were called by the police who said they were taking the patient to hospital as he seemed unwell – they did not tell you more about this on the phone.

You found out from the patient that the police deployed a taser and are very annoyed about this, as the patient is never angry or aggressive. You would like the doctor to make the shaking stop and find out if the taser caused it.

# Scenario 2 Instructions for doctor

## **Summary**

You have been asked to see a patient in the Emergency Department who has sustained a head injury following a fall. The patient is currently stable and is yet to be seen.

## **Instructions to doctor**

- Assess the patient and elicit their concerns
- Form an effective plan for the patient and identify any risks

# Scenario 2 Instructions for ACTOR

## Summary

**Patient:** You are a 33 female patient who is 19 weeks pregnant and have come to Emergency Department due to an injury to your nose and right cheek. Due to the pain, you want to check there is no fracture. You have told the nurse at triage that the injury was due to a fall at home, and that you have been getting dizzier with the pregnancy. You said that you got dizzy in your last pregnancy too and aren’t too worried about this.

In truth, you did not have a fall, but instead you have been experiencing domestic abuse at home and were assaulted by your partner. You partner was also physically abusive during your last pregnancy, but you have not received any support for this and are not sure what support you could access.

Since the start of the pregnancy, you’ve noticed that your partner always seems to know where you are and who you’ve been talking with. You’re concerned that he is in some way tracking your phone, as he always seems to know if a friend is messaging you being concerned.

You won’t talk to the doctor if your phone is turned on in the room due to your worries that it’s being listened too. If the doctor notices your phone ringing and suggests turning it off, or leaving it outside, you say yes to this if they think it’s a good idea.

Only if the phone is off, or outside the room, are you willing to talk about the physical abuse. If you do have this discussion, you are willing to access support however you don’t know how to do this with your partner watching your phone.

# Scenario 3 Instructions for doctor

## **Summary**

Your next patient is a teenager who has come in with their Mum and Dad who are both very concerned about a problem with his hand. The nurse has told you that they are talking about a microchip or some other implanted technology.

## **Instructions to doctor**

- Take a history from the patient and identify their concerns
- Discuss options with the patient and their parents, and form a management plan.

# Scenario 3 Instructions for ACTOR

## Actor 1 = Patient

## Actor 2 = Patient’s Mum

## Actor 3 = Patient’s Dad

## Summary

**Patient:** You are a 16-year-old male teenager who recently found out about RFID Chips (small biometric implants) that you can get implanted under the skin in the back of your hand. You found out about them online and heard that they were similar to getting a tattoo, but you could use them for other cool things like opening doors and making payments. You attended a body modification clinic in London which provides the implants, and had one put in your right hand for £100. The operation itself only took 30minutes, involved a small needle to give some numbing liquid and the chip was injected.

Now that you have the implant in, you can go back to the clinic and have it programmed to match your ID badge e.g. your badge for school or the gym, and you can swipe in with your hand instead. You got the implant because you think it’s fun and cool, but you want to make sure you can still play basketball with it in place.

**Patient’s Mum:** You are very concerned about this new technology which you have not heard about before – you found out about it from the mum of one of your son’s friends. You’ve been reading about implanted microchips online and are worried that they can have all kinds of complications, and that they produce dangerous radiation. You want the chip taken out now and you don’t think your son should be allowed to have one.

**Patient’s Dad:** You are not that worried about the chip and think that it’s just a silly party trick – as far as your concerned, it’s similar to the ones that vets put in cats. You do think its important that your son can still play basketball, so would like to know if that would be a problem.

# Scenario 4 Instructions for doctor

## **Summary**

You’ve been asked to see a patient complaining of left arm pain which is associated with some loss of motor function and sensation.

## **Instructions to doctor**

- Take a history from the patient and identify their concerns
- Discuss options with the patient and their parents, and form a management plan.

# Scenario 4 Instructions for ACTOR

## Summary

**Patient:** You have stared experiencing pain in your left arm over the past week, which is now predominantly affecting your left hand. The pain is associated with tingling (‘pins and needles’) and numbness, in the distribution presented in Figure 1 below. As a result of the symptoms, you are now struggling to write, type, cook and clean.

There is no injury that preceded the problem, and you can’t think of any possible cause. You do not have any history of problems to the limb and no underlying problems with your bones or joints.

In terms of your past medical history, you had an Implantable Cardioverter Defibrillator (ICD) that was placed 5 years earlier following a sudden cardiac arrest thought to be related to Brugada’s syndrome. You remember this being very scary when it happened, all of a sudden you had chest pain and felt unwell – you were told you’d had a cardiac arrest afterwards due to an underlying problem with your heard. However, since having the ICD you’ve never had any further issues.

Your regular medications include Vitamin D and Iron Supplements, which aren’t prescribed, you buy them over the counter.

Today, you would like the doctor to find the cause of your symptoms and help stop the pain.

**Figure 1: Distribution of pain and numbness.**


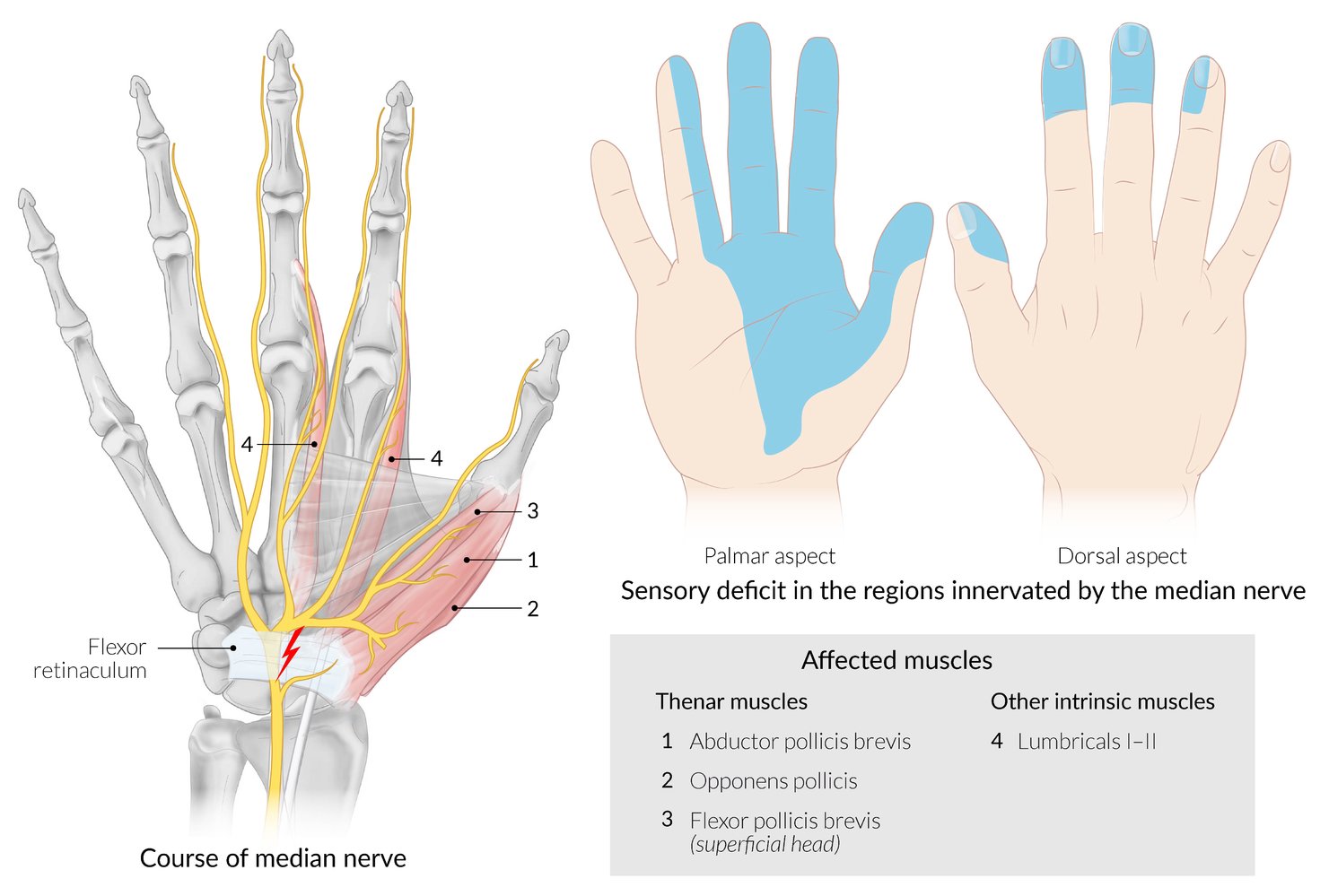

Supplement: sj-docx-2-dhj-10.1177_20552076241247939 - Supplemental material for Simulation-based research for digital health pathologies: A multi-site mixed-methods study [file sj-docx-2-dhj-10.1177_20552076241247939.docx]
